# Development of a prognostic model for children with neuroblastoma based on necroptosis-related genes

**DOI:** 10.3389/fgene.2022.947000

**Published:** 2022-08-05

**Authors:** Jing Chu

**Affiliations:** Department of Pathology, Anhui Provincial Children’s Hospital, Hefei, China

**Keywords:** neuroblastoma, tumor microenvironment, necroptosis, risk score, immunotherapy

## Abstract

**Background:** Neuroblastoma (NBL) is a rare malignant tumor of the peripheral sympathetic nervous system in children with a low overall survival rate. Recent studies have revealed the important role of necroptosis in the occurrence and development of many kinds of tumors. In this study, a prognostic model based on necroptosis-related genes was constructed for NBL.

**Methods:** Expression profiles and clinical information for patients with NBL were downloaded from TARGET. Data for necroptosis-related genes were extracted for Cox regression and lasso regression analyses to evaluate factors associated with prognosis and to construct a prognostic model. Data from the GEO datasets GSE62564 and GSE85047 were used for external verification. Associations between risk scores were calculated, and immune infiltration, drug sensitivity, and mutation analyses were conducted. Functional enrichment analyses of genes in the prognostic model were performed.

**Results:** Six necroptosis-related genes (i.e*., CYLD, JAK1, APC, ERH, CNBP*, and *BAX*) were selected to construct a prognostic risk model. The risk score was highly correlated with levels of infiltration of multiple immune cells and sensitivity to common antineoplastic drugs. In addition, the risk score was identified as an independent prognostic factor for patients with NBL.

**Conclusion:** We constructed and validated a prognostic model based on necroptosis-related genes, providing insights into the development and progression of NBL and a basis for improved management. In addition to providing a tool for clinical decision-making, these findings support the importance of necroptosis in NBL and may guide the development of therapeutic strategies targeting this process.

## Introduction

Neuroblastoma (NBL) is a malignant tumor of the peripheral nervous system originating from primitive neural crest cells, accounting for 8%–10% of all malignant tumors and approximately 15% of tumor-related deaths in children. The morbidity is slightly higher in boys than in girls (ratio 1.2:1). The incidence peaks at 0–4 years of age, with a median age of 23 months (Park et al., 2010). NBL shows clinical and biological heterogeneity, and the disease spectrum ranges from spontaneous regression under no medical intervention or differentiation to an aggressive state with treatment resistance and tumor metastasis, despite intensive treatment. Therapy based on risk stratification by clinicopathological (diagnostic age, clinical staging, and histopathology) and genetic factors (MYCN amplification) significantly improves prognosis in low- and medium-risk patients, with 5-years survival rates ranging from 70% to 98%. However, about 50% of patients have high-risk characteristics with a 5-years survival rate after diagnosis of less than 40% (Fusco et al., 2018). Therefore, a comprehensive understanding of the pathogenesis of NBL, biomarker identification, and the development of an effective prognostic model are of great significance for improving outcomes in NBL.

Programmed cell death is a natural barrier to the occurrence and development of cancer and can be classified as apoptotic and non-apoptotic, including ferroptosis, pyroptosis, autophagy, and necroptosis (Dai et al., 2020). Evasion and resistance to programmed cell death are acquired by cancer cells (Hanahan and Weinberg, 2011). Resistance to apoptosis is an important cause of chemotherapeutic drug resistance in patients with cancer (Johnstone et al., 2002). Therefore, it is imperative to develop methods to induce non-apoptotic forms of programmed cell death as alternative therapeutic approaches. Necroptosis is a recently caspase-independent mechanism of cell death. It is mainly mediated by receptor-interacting protein kinase-1 (RIPK1) and -3 (RIPK3) and their target, mixed lineage kinase domain-like (MLKL). It is related to a variety of human diseases, including ischemia-reperfusion injury, inflammation, allograft rejection, neurodegenerative diseases, autoimmune diseases, and cancer (Negroni et al., 2020). Necroptosis plays dual roles in cancer development. On the one hand, adaptation to necroptosis in the tumor microenvironment promotes metastasis, suggesting that the inhibition of necroptosis is an anti-metastasis strategy. On the other hand, the expression levels of key mediators of necroptosis in some cancers are downregulated, suggesting that necroptosis has anticancer effects (Najafov et al., 2017).

However, the prognostic value of necroptosis-related genes in children with NB has not been evaluated. In this study, the association between necroptosis-related genes and prognosis in NB was evaluated and a prognostic model was constructed. These findings provide insight into the prognostic value of genes related to necroptosis and preliminarily uncover the complex biological functions and immunoregulatory effects of these genes and their regulatory networks.

## Materials and methods

### Data acquisition

The TARGET database (https://portal.gdc.cancer.gov/), as the largest cancer gene information database, stores data for gene expression, miRNA expression, copy number variation, DNA methylation, single nucleotide polymorphisms, and so on. Processed raw mRNA expression data for NBL were downloaded, including data for 158 NBL samples. The Series Matrix File for GSE62564 was downloaded from NCBI GEO and the annotation platform was GPL11154. Data for 495 patients with NBL with complete expression profiles and survival information were extracted. The Series Matrix File for GSE85047 whose annotation platform was GPL5175 was obtained. Data for 275 patients with NBL with complete expression profiles and survival information were retrieved. A total of 604 gene sets including necroptosis-related genes were obtained from the GeneCards database (https://www.genecards.org/).

### Gene ontology and encyclopedia of genes and genomes functional annotation

Prognostic genes were annotated using clusterProfiler (R3.6) to thoroughly explore their functions. Gene Ontology (GO) and Kyoto Encyclopedia of Genes and Genomes (KEGG) enrichment analyses were performed; terms and pathways with *P*- and *Q*-values of less than 0.05 were considered significant.

### Metascape

Metascape (http://metascape.org/) is a powerful analytical tool for functional annotation of genes and proteins that allows users to apply current popular bioinformatics methods to batch gene and protein analyses to achieve an understanding of gene or protein function. GO/KEGG functional annotation of genes was performed using the Metascape database. Minimum overlap ≥3 and *p* ≤ 0.05 were considered to be significant.

### Model construction and prognosis

Necroptosis-related genes were selected, and a univariate Cox proportional hazards regression model was applied. Necroptosis genes with *p* < 0.05 were considered statistically significant and included in the subsequent analysis. Lasso penalized Cox regression analysis was performed using 10-fold cross-validation based on the “glmnet” package in R to further reduce the number of necroptosis genes with the best predictive performance in the selected panels. After including the expression values for each specific gene, a risk score formula for each patient was constructed and weighted by its estimated regression coefficients in the lasso regression analysis. According to the risk score formula, the patients were divided into a low-risk group and a high-risk group with the median risk score value as the cut-off point. Differences in survival between the two groups were assessed by Kaplan-Meier analysis and compared using the log-rank statistical method. Lasso regression and stratified analyses were performed to examine the role of risk scores in the prediction of patient outcomes. The “survivalROC” package was used to derive receiver operator curves (ROCs) to investigate the accuracy of the model predictions. Univariate and multivariate Cox analyses including age, sex, tumor stage, and necroptosis score were performed to identify independent prognostic factors.

### Drug sensitivity analysis

Based on the largest pharmacogenomics database (GDSC Cancer Drug sensitivity Genomics Database, https://www.cancerrxgene.org/), the R package “pRRophetic” was used to predict the chemosensitivity of each tumor sample. The estimated IC50 values for each specific chemotherapeutic drug were obtained by regression, and the prediction accuracy was measured by 10-fold cross-validation with the GDSC training set. Default values were selected for all parameters, using “combat” to remove the batch effect and the average value of repeated gene expression estimates.

### Analysis of immune cell infiltration

The RNA-seq data for patients with NBL in different subgroups were analyzed by the CIBERSORT algorithm to infer the relative proportions of 22 kinds of immune-infiltrating cells. A Spearman correlation analysis was used to analyze the risk score and levels of infiltrating immune cells. Results were considered statistically significant at *p* < 0.05.

### Gene set variation analysis

A gene set variation analysis (GSVA) is a non-parametric and unsupervised method to evaluate gene enrichment. By comprehensively scoring the set of genes of interest, GSVA converts gene level changes into pathway level changes to gain insight into biological functions. In this study, gene sets were downloaded from Molecular Signatures Database (v7.0), and each gene set was scored by the GSVA algorithm to evaluate differences in biological functions between samples.

### Gene set enrichment analysis

A gene set enrichment analysis (GSEA) was performed with predefined gene sets to rank genes according to the degree of differential expression between two groups of samples and to determine whether a predefined gene set was enriched. GO terms and KEGG signaling pathways were obtained for differentially expressed genes between the high-risk group and low-risk group by GSEA; the number of replacements was set to 1,000 and the type of replacement was set to phenotype.

### Regulatory network analysis of prognostic genes

Cistrome DB is a comprehensive database for ChIP-seq and DNase-seq analyses, containing data for transcription factors, histone modifications, and chromatin accessibility of 30,451 human and 26,013 mouse samples. The regulatory relationships between transcription factors and genes in the prognostic model were evaluated using Cistrome DB, in which the genome file was set to hg38 and the transcription initiation site was set to 10 kb. The results were visualized using Cytoscape.

### Immunohistochemical staining analysis

To verify the protein expression of the necroptosis-related genes, ganglioneuroma samples were chosen as a control group, and IHC staining was used to evaluate the expression of these genes in paraffin-embedded tissues in the NBL group and control group. The paraffin-embedded serial tissue sections were cut at a thickness of 4 μm, and IHC was used to detect CYLD, JAK1, APC, ERH, CNBP, and BAX. The SP method was used to conduct IHC, and the primary antibodies against CYLD, JAK1, APC, ERH, CNBP, and BAX were all purchased from Abcam (Cambridge, United Kingdom). All experiments were carried out at least three times independently. Pannoramic SCAN (3DHISTECH, Budapest, Hungary) was used for observations and to obtain images. Image Pro Plus 6.0 was used to analyze the IHC results.

### Statistical analysis

Survival curves were generated by the Kaplan-Meier method and compared by the log-rank test. A Cox proportional risk model was used for multivariate analyses. All statistical analyses were performed using R (version 3.6). All statistical tests were two-sided, and results were considered statistically significant at *p* < 0.05.

## Results

### Expression of necroptosis-related genes in NBL

The processed raw mRNA expression data for NBL in the TARGET database (FPKM) were downloaded, and necroptosis-related gene sets were obtained using the GeneCards database. We used clinical information for patients with NBL for a Cox univariate regression analysis to screen for necroptosis-related genes associated with prognosis in NBL. The following 14 prognosis-related genes were filtered (*p* < 0.001) by Cox univariate regression (in decreasing order based on significance): *BAX, ERH, CYLD, CPSF3, JAK1, EMD, EIF4EBP1, ATAD3A, HNRNPF, ADRM1, FUS APC, CCT5,* and *CNBP* (Table 1).

**TABLE 1:** Expression of necroptosis-related genes in NBL.

Gene	HR	z	*p*-value	Lower	Upper
BAX	1.98371619284125	5.48987193560628	4.02225271441093e-08	1.55337039660584	2.53328500552022
ERH	1.85233482253478	4.92782242546772	8.31511352013574e-07	1.44956691399528	2.36701338975658
CYLD	0.517434644838194	−4.34424835249258	1.3975334136114e-05	0.384377274471897	0.696551615978544
CPSF3	1.61988040244062	4.14992974066646	3.32577342461729e-05	1.28987185896012	2.03432030862867
JAK1	0.578925385579156	−3.77867763652999	0.00015766336556394	0.436010753947224	0.76868425614232
EMD	1.60016941917157	3.72777940585821	0.000193174345858099	1.24974414578669	2.04885310220044
EIF4EBP1	1.44851990579632	3.64660523798732	0.000265727677537716	1.24974414578669	2.04885310220044
ATAD3A	1.54022966941195	3.57903960381036	0.000344859209857309	1.21579227879252	1.95124403725686
HNRNPF	1.52677719453705	3.48401010645772	0.000493960835195072	1.20334696141417	1.93713756423083
ADRM1	1.49234932554181	3.48123248071431	0.00049911208043814	1.19118688309065	1.86965331893737
FUS	1.53804846136142	3.47146778172561	0.000517621441105841	1.20616906533564	1.96124501737073
APC	0.591730613045381	−3.47129043850349	0.000517963442372491	0.440007876948232	0.795770114034248
CCT5	1.56674940484423	3.45305994008363	1.21427595692257	1.21427595692257	2.0215369361351
CNBP	1.58200627442629	3.42348400836818	1.21663520141189	1.21663520141189	2.05710294213068

### Functional enrichment of prognosis-related genes and construction of protein interaction networks

**FIGURE 1:**
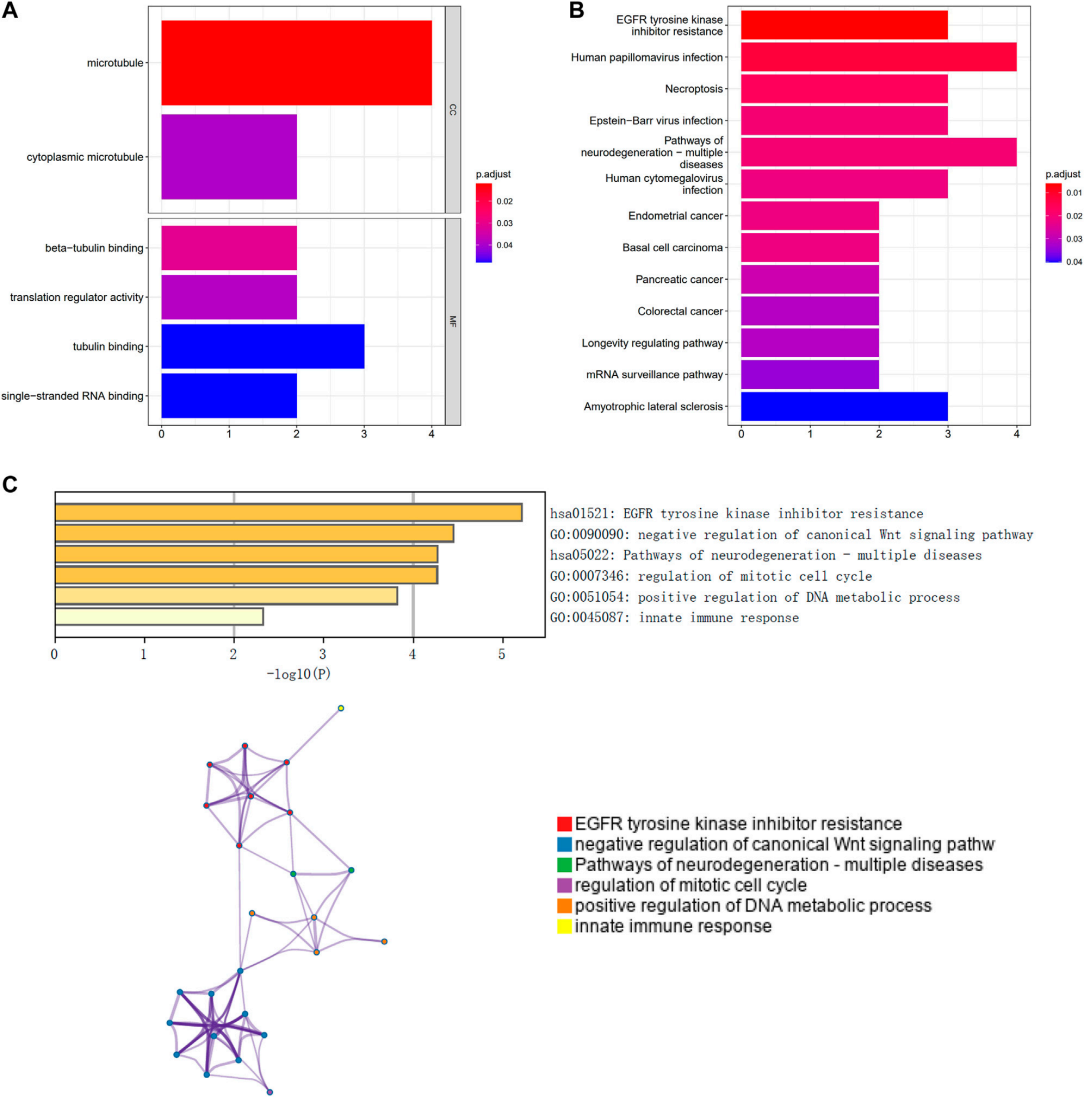
Functional enrichment of prognosis-related genes and construction of protein interaction networks. **(A)** GO analysis; **(B)** KEGG pathway analysis; **(C)** Metascape functional analysis.

GO and KEGG pathway enrichment analyses revealed that the prognostic genes were significantly enriched in a large number of pathways. Enriched GO terms included cytoplasmic microtubule and translation regulator activity (Figure 1A). Enriched KEGG pathways included Human papillomavirus infection and Basal cell carcinoma (Figure 1B). A functional analysis using Metascape revealed that these prognostic genes were also highly enriched in several related pathways (Figure 1C).

### Prognostic model construction and internal validation of necroptosis-related genes

**FIGURE 2:**
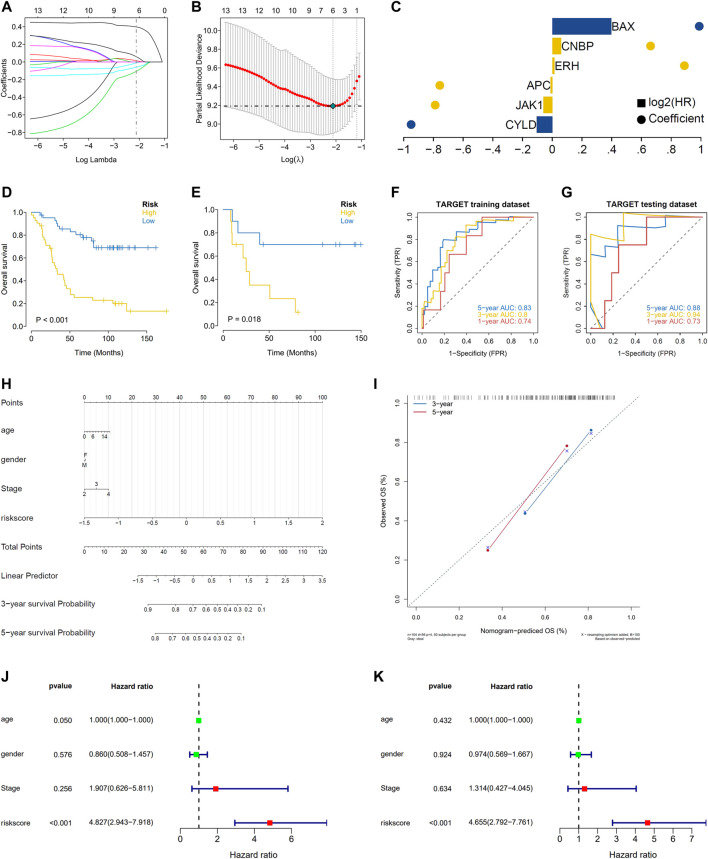
Lasso analysis and model construction. **(A)** LASSO coefficient profiles of the 13 IRGs in TARGET-NBL; **(B)** A coefficient profile plot was generated against the log (lambda) sequence. Selection of the optimal parameter (lambda) in the LASSO model for TARGET; **(C)** Lasso Coefficient HR; **(D)** Kaplan-Meier survival curve analysis in the high-risk and low-risk NBL patients in the training subset; **(E)** Kaplan-Meier survival curve analysis in the high-risk and low-risk NBL patients in the testing subset; **(F)** time-dependent ROC curve for 1-year, 3-years, and 5-years prediction (training subset); **(G)** time-dependent ROC curve for 1-year, 3-years, and 5-years prediction (testing subset); **(H)** the nomogram for predicting the 3- and 5-years OS of NBL patients; **(I)** the calibration curve of the nomogram for predicting 3- and 5-years OS of NBL patients; **(J)** Univariate Cox regression analyses in the TARGET cohort NBL patients; **(K)** multivariate Cox regression analyses in the TARGET cohort NBL patients.

We randomly divided patients from the TARGET database into training and validation sets at a ratio of 4:1. We obtained the risk score value for each sample based on the model obtained by lasso regression (Risk Score = *CYLD* × (−0.105492134) + *JAK1* × (−0.059871886) + *APC* × (−0.0109496) + *ERH* × 0.014856456 + *CNBP* × 0.059824071 + *BAX* × 0.39721508) (Figures 2A–C). Patients were divided into high-risk and low-risk groups using the median risk score as a threshold for analyses by Kaplan-Meier curves. Overall survival (OS) was significantly lower in the high-risk group than in the low-risk group in both the training set and the test set (Figures 2D,E). In addition, an ROC curve analysis showed that the AUC values in the training set and the test set for periods of 1, 3, and 5 years were all greater than 0.70 (Figures 2F,G), suggesting that the model was effective.

We integrated the clinical information as well as risk scores for patients in the high- and low-risk groups for regression analyses. A logistic regression analysis showed that in all of our samples, the distribution of values for multiple clinical indicators and risk scores for pediatric NBL contributed to multiple scoring processes. Age, gender, stage, and risk scores were evaluated with respect to 3-years and 5-years OS (Figure 2H). We also corrected the predicted OS in NBL for two periods of 3 and 5 years (Figure 2I). The risk score was identified as an independent prognostic factor for NBL by univariate and multivariate analyses (Figures 2J,K).

### Multi-omics analysis of the clinical predictive value of the model

**FIGURE 3:**
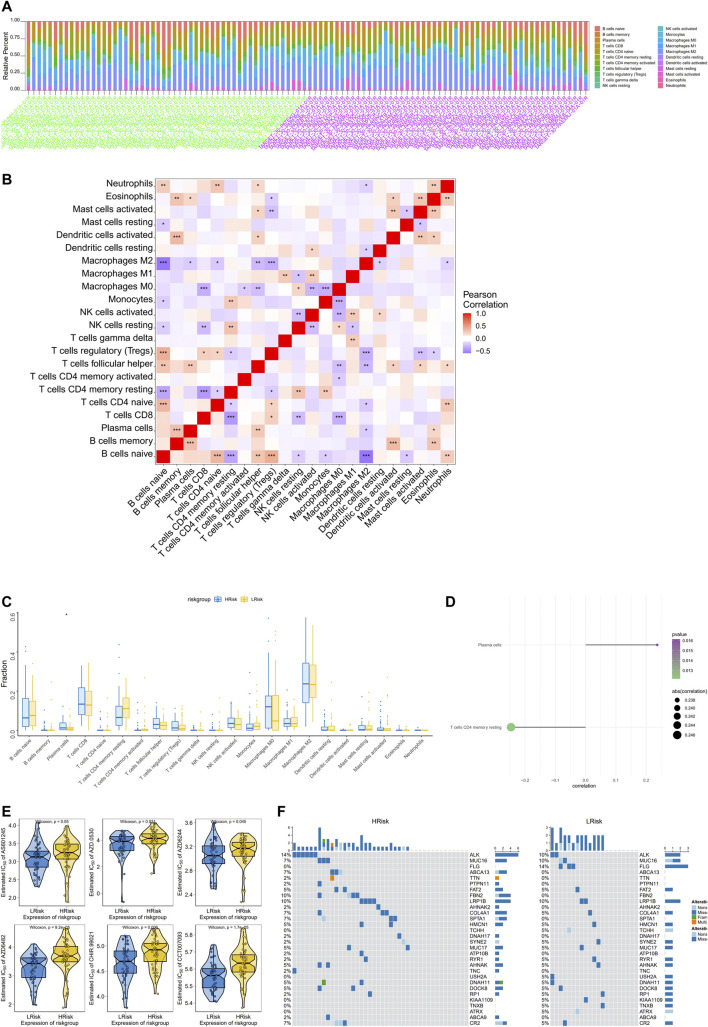
Multi-omics analysis of the clinical predictive value of the model. **(A)** Stacked bar chart of the distribution of 22 immune cells in each NBL sample of the TARGET cohort; **(B)** Pearson correlation between immune cells, red for positive correlation, purple for negative correlation; **(C)**Differences in immune cell counts between the high-risk and low-risk groups; **(D)**The correlation between the risk score and the immune cells, the circle size indicates the strength of the correlation, and the color indicates the *p*-value; **(E)** The difference on the therapeutic sensitivities of six chemotherapy drugs; **(F)** Mutation profiles of patients in the high and low risk groups, yellow for high risk group and blue for low risk group.

The tumor microenvironment is mainly composed of tumor-associated fibroblasts, immune cells, extracellular matrix, various growth factors, inflammatory factors, specific physical and chemical characteristics, and cancer cells. The tumor microenvironment significantly affects tumor diagnosis, survival outcomes, and sensitivity to therapies. By analyzing the relationship between the risk score and tumor immune cell infiltration, we further explored the molecular mechanisms by which the risk score affects NBL progression. The distribution of levels of infiltration of different immune cell types in the samples differed between groups (Figure 3A). Correlations were detected between the risk score and multiple cell types in the tumor microenvironment (Figure 3B). Additionally, levels of plasma cells were significantly lower in the low-risk group than in the high-risk group (Figure 3C). The risk score was significantly correlated with plasma cells and CD4 memory resting T cells (Figure 3D). Drug sensitivity data were obtained from the GDSC database, and the R package “pRRophetic” was used to predict the sensitivity of each tumor sample to chemotherapy. The risk score was significantly associated with sensitivity to various drugs, including AS601245, AZD.0530, AZD6244, AZD6482, CHIR.99021, and CCT007093 (Figure 3E). We further explored the mutation profiles of patients in the high- and low-risk groups. The mutation frequency in genes, such as *ALK,* was significantly higher in the high-risk group than in the low-risk group (Figure 3F).

### Exploration of specific signaling mechanisms associated with prognostic models

**FIGURE 4:**
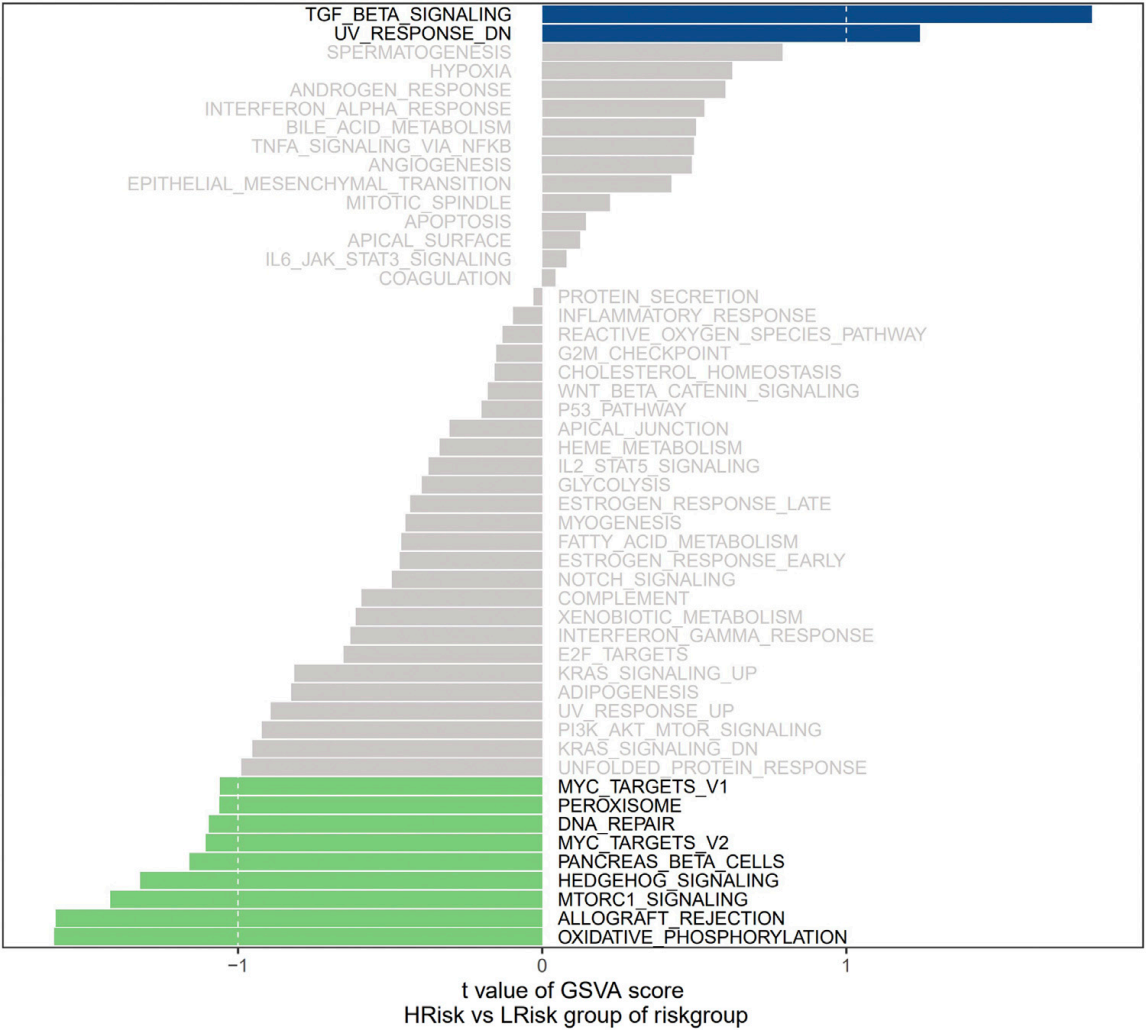
GSVA plot of riskgroup.

We next investigated the specific signaling pathways related to a high and low risk to explore the molecular mechanisms by which risk scores influence tumor progression. The results of GSVA showed that the enriched differential pathways between the two groups were mainly TGF BETA SIGNALING, UV RESPONSE DN, MTORC1 SIGNALING, ALLOGRAFT REJECTION, and OXIDATIVE PHOSPHORYLATION (Figure 4), suggesting that perturbations in these signaling pathways in patients in the high- and low-risk groups affected prognosis in NB.

### Validation of the robustness of the prognostic model using an external dataset

**FIGURE 5:**
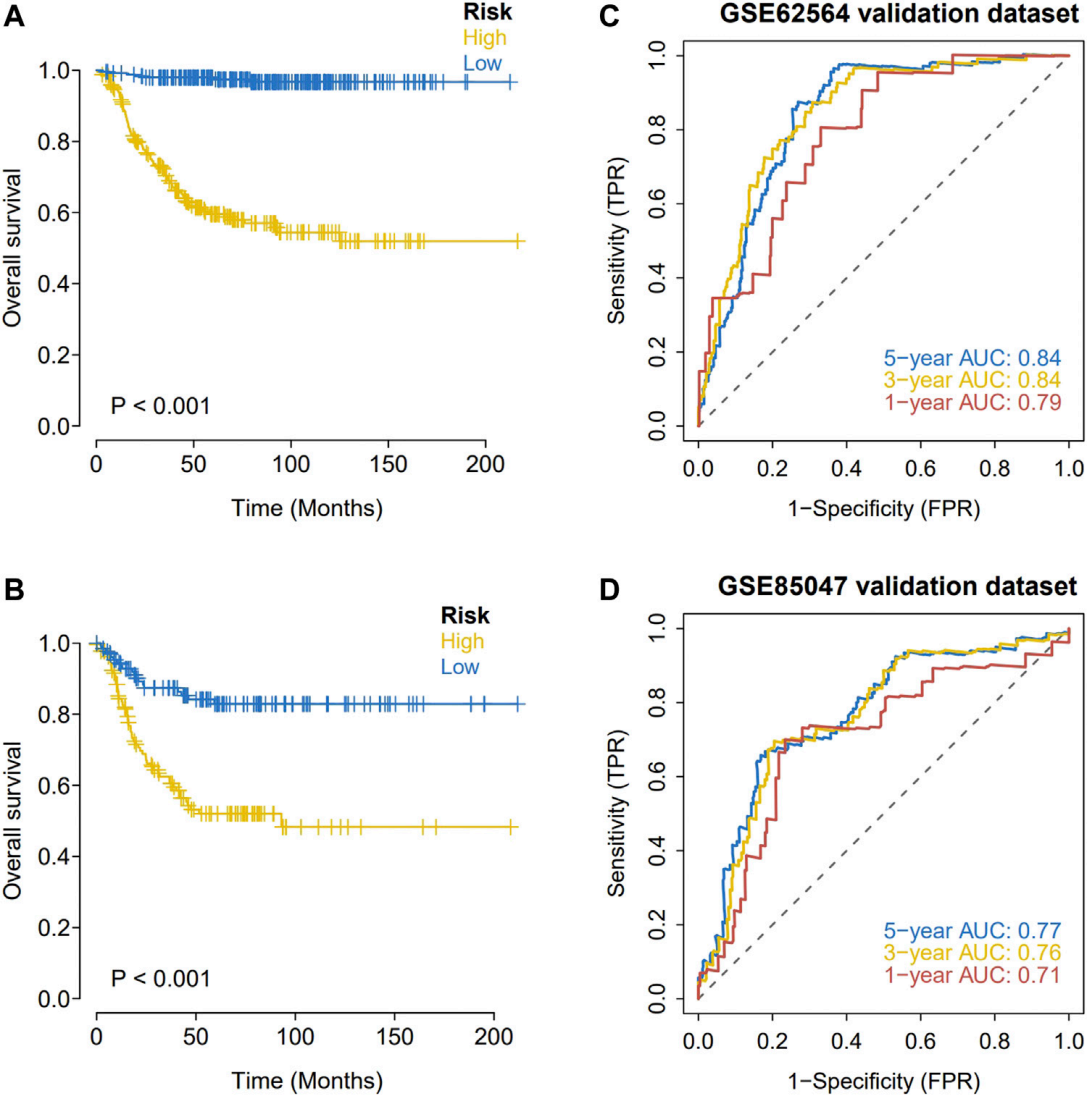
External validation of the hypoxia risk score. **(A,B)** Validation of the hypoxia risk score in GSE62564; **(C,D)** Validation of the hypoxia risk score in GSE85047.

Expression data and survival information for patients with NBL were downloaded from the GEO database (GSE62564 and GSE85047) to predict the clinical stage based on the model and for a Kaplan-Meier analysis of the survival difference between groups. In the two GEO external validation sets, OS was significantly lower in the high-risk group than in the low-risk group (Figures 5A,B). As determined by ROC curve analyses, the model had strong predictive power for prognosis (AUC values for 1 year, 3 years, and 5 years with the GEO verification data set were all greater than 0.70) (Figures 5C,D).

### Signaling mechanisms associated with the prognostic model

**FIGURE 6:**
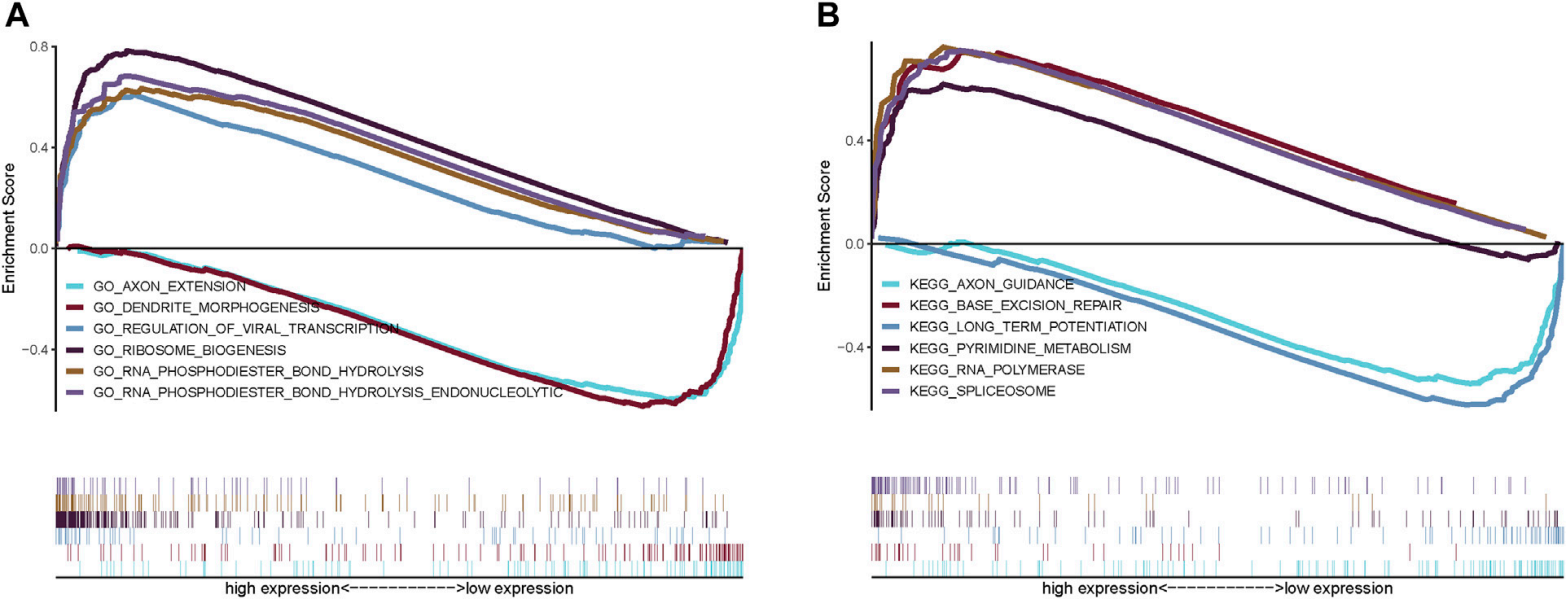
GSEA analysis of risk scores. **(A)** GO pathways; **(B)** KEGG pathways.

We next investigated the specific signaling pathways differentiating samples in the high- and low-risk groups based on the genes in the prognostic models to evaluate the factors contributing to tumor progression. By a GSEA, we found significant enrichment for many related pathways, including the GO terms AXON EXTENSION, DENDRITE MORPHOGENESIS, and REGULATION OF VIRAL TRANSCRIPTION and the KEGG pathways BASE EXCISION REPAIR, LONG TERM POTENTIATION, and AXON GUIDANCE (Figures 6A,B), suggesting that the disturbance of these signaling pathways in high and low risk groups affected prognosis in NBL.

### Relationships between expression levels of genes in the prognostic model and immune cell infiltration

**FIGURE 7:**
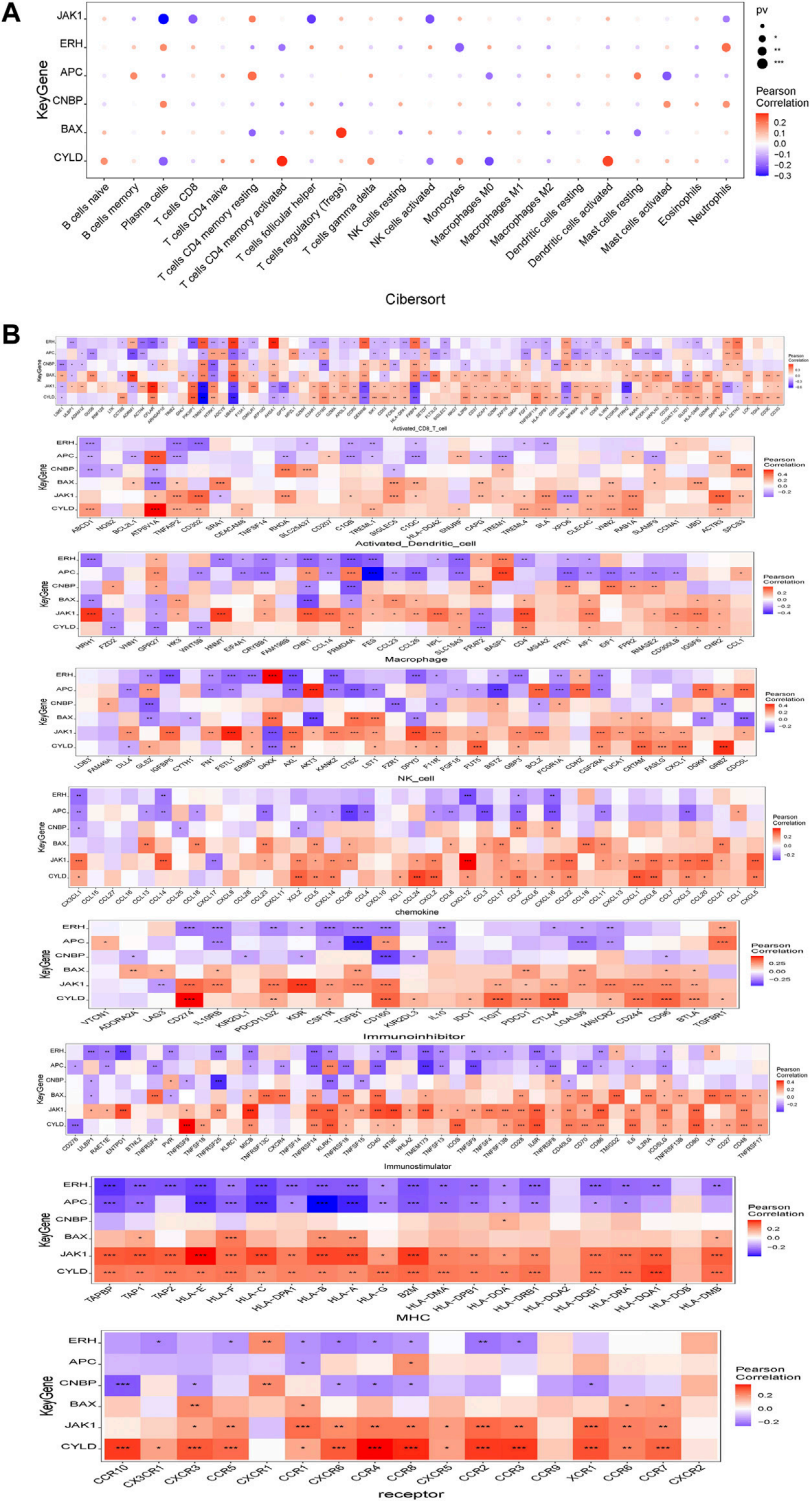
Relationship between model gene and immune infiltration. **(A)** Pearson correlation between model genes and 22 kinds of immune cells; **(B)** Pearson correlation analysis between model genes and various immune-related genes. *means *p* < 0.05; ** means *p* < 0.01; *** means *p* < 0.001.

Several genes in the prognostic model were highly correlated with levels of infiltrating immune cells. For example, *BAX* was positively correlated with regulatory T cells (Tregs) and plasma cells and negatively correlated with CD4 memory resting T-cells and resting mast cells. *ERH* was positively correlated with plasma cells and neutrophils and negatively correlated with CD4 memory activated T cells and monocytes. *CYLD* was positively correlated with CD4 memory activated T cells and activated dendritic cells and negatively correlated with plasma cells and M0 macrophages. *JAK1* was positively correlated with CD4 memory resting T cells and activated dendritic cells and negatively correlated with follicular helper T cells and plasma cells. *APC* was positively correlated with memory B cells and CD4 memory resting T cells and negatively correlated with M0 macrophages and activated mast cells. *CNBP* was positively correlated with plasma cells and activated mast cells and negatively correlated with CD4 memory activated T cells and Tregs (Figure 7A). We further evaluated the correlations between genes in the prognostic model and immune factors, including immunomodulators, chemokines, and cellular receptors, using TISIDB (Figure 7B). These analyses confirmed that the prognostic genes are closely related to levels of immune cell infiltration and play important roles in the immune microenvironment.

### Regulatory network analysis

**FIGURE 8:**
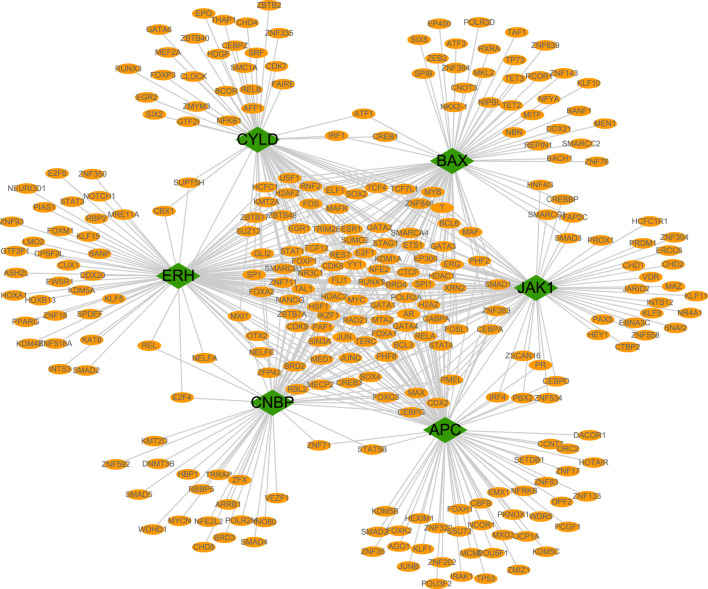
Transcriptional regulatory networks of model genes. The transcriptional regulatory network of core genes was based on cistrome database, and the species was set as HG38, and the transcription start site was set as 10 KB. Green represents model genes and orange represents associated transcription factors.

We evaluated the transcriptional regulatory network of the six genes in the prognostic model. Using Cistrome DB, 91 transcription factors were related to *BAX*, 98 were related to *ERH*, 78 were related to *CYLD*, 85 were related to *JAK1*, 10 were related to *APC*, and 68 were related to *CNBP*. The results were visualized using Cytoscape to obtain a comprehensive transcriptional regulatory network involving genes in the prognostic model (Figure 8).

### ceRNA network analysis

**FIGURE 9:**
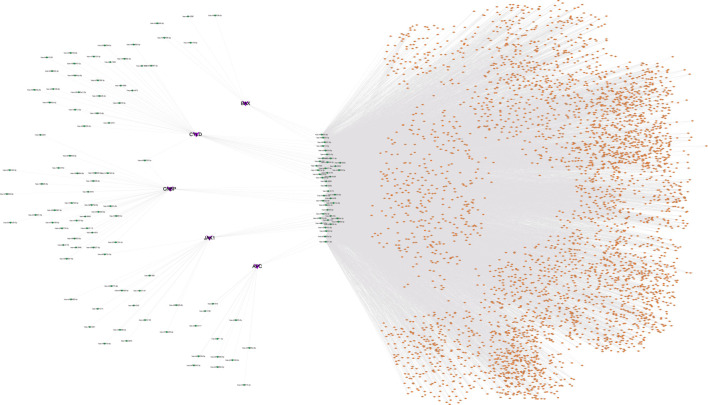
Model gene-related ceRNA network, purple represents mRNA, green represents miRNA, yellow represents lncRNA. Based on miRWalk, TargetScan and miRDB databases, using Cytoscape software visualization.

The six genes in the prognostic model were analyzed using the miRWalk and ENCORI databases to predict interacting miRNAs and lncRNAs, respectively. Interacting mRNA–miRNA pairs associated with these six key mRNAs were first extracted using the miRWalk database, and only 605 mRNA–miRNA pairs with a TargetScan score of one or miRDB score of one were retained, involving 5 mRNAs and 131 miRNAs. Then, the interacting lncRNAs were predicted based on these miRNAs. A total of 18,244 pairs of interactions were predicted (involving 42 miRNAs and 3,868 lncRNAs). Finally, we constructed the ceRNA network using Cytoscape (v3.7) (Figure 9).

### Immunohistochemical staining analysis

**FIGURE 10:**
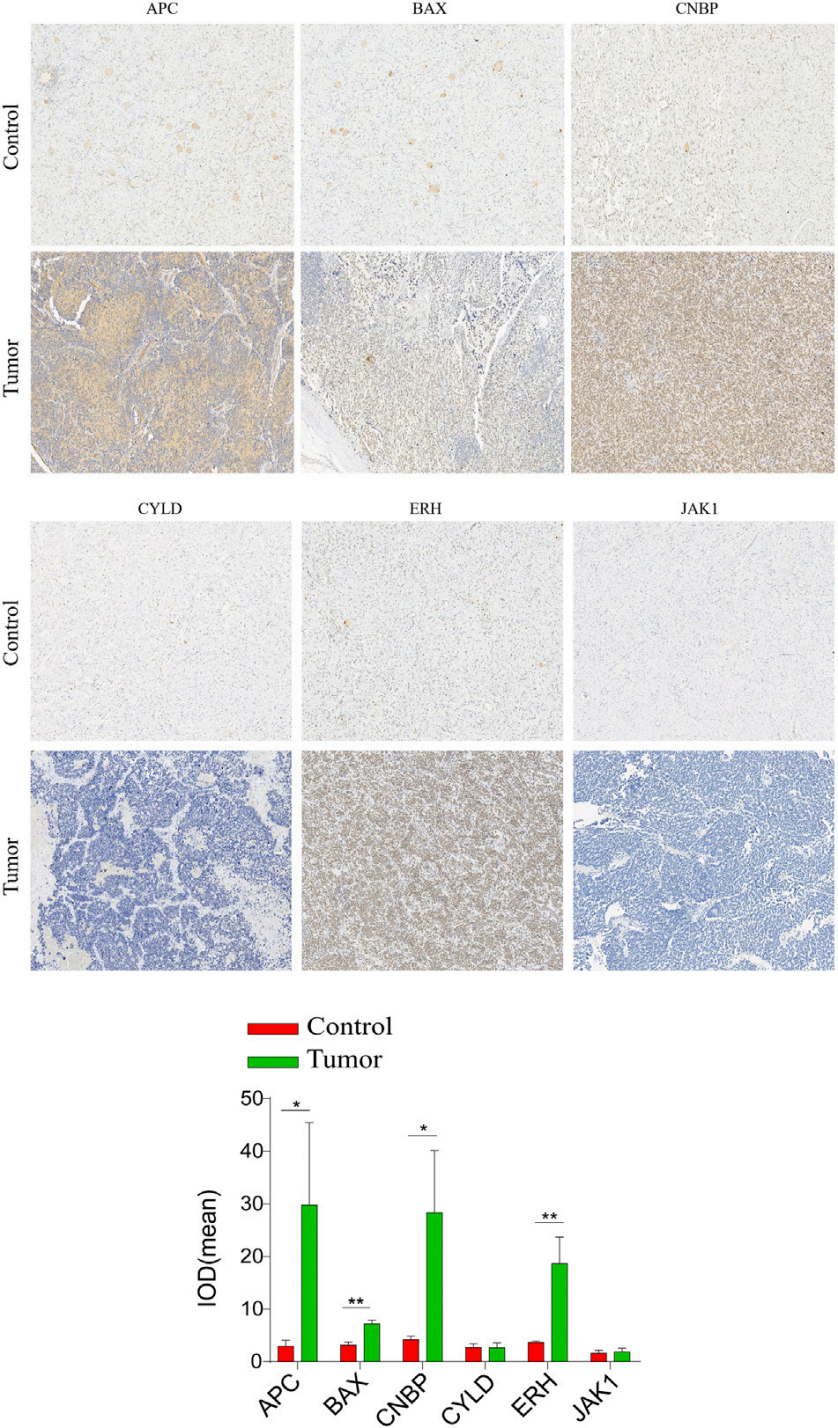
Validation of the expression of the prognostic Necroptosis in NBL and Ganglioneuroma (GN) by immunohistochemical staining analysis. The expressions of CYLD、JAK1、APC、ERH、CNBP, and BAX in NBL tissues and GN were detected by immunohistochemical staining (magnification ×100). Quantification of immunohistochemical staining for CYLD、JAK1、APC、ERH、CNBP, and BAX by Image Pro Plus software. *p < 0.05 vs. control group, **p < 0.01 vs. control group.

IHC assays were performed to verify the expression levels of proteins encoded by these necroptosis-related genes in the NBL and ganglioneuroma (control) groups. As shown in Figure 10, the expression levels of BAX (*p* = 0.0017), ERH (*p* = 0.0067), APC (*p* = 0.0416), and CNBP (*p* = 0.0244) were significantly higher in tumor tissues of the NBL group than in the control group. Other indexes did not differ significantly between groups.

### Disscussion

Necroptosis has conflicting roles in malignant tumors, with both tumor-promoting and inhibitory effects in different types of adult cancers. In particular, necroptosis can inhibit tumor progression but can also trigger inflammatory responses and vascular endothelial cell necrosis, in turn promoting tumor cell extravasation and cancer metastasis (Gong et al., 2019). The delayed activation or disruption of normal apoptotic pathways may be an important cause of chemotherapeutic drug resistance in patients with NBL; therefore, the induction of necroptosis in NBL may be an alternative therapeutic approach to eliminate anti-apoptotic tumor cells and improve the anti-tumor immune microenvironment.

In this study, we constructed the first prognostic model for NBL based on necroptosis-related genes. We first obtained information on necroptosis-related genes from the GeneCards database and used univariate Cox regression and LASSO regression analyses to screen for necroptosis-related genes associated with prognosis, revealing six genes, i.e*., CYLD, JAK1, APC, ERH, CNBP*, and *BAX*, which were used to construct a prognostic risk model. The conserved cylindromatosis (CYLD) is a deubiquitinating (DUB) enzyme with an important regulatory role in a variety of cellular processes, including the immune response, inflammation, and necrosis. Small ubiquitin-related modifier (SUMO) can post-translationally modify CYLD to impair its DUB function. After 8 days of treatment with all-trans-retinoic acid (ATRA) on the NB SK-N-BE 2) C cell line, the SUMOization of CYLD decreased, while its expression increased, which blocked the NF-κB signal transduction pathway and promoted cell death (Kobayashi et al., 2015). In prostate cancer cell lines, the knockout of *CYLD* increased the proliferation, migration, colony formation, and invasion of cancer cells *in vitro* (Haq et al., 2022). JAK1 (Janus kinase 1) is a member of a class of protein-tyrosine kinases involved in autoimmune diseases and malignancies. The targeted inhibition of JAK1 expression by miR-20a-5p can decrease proliferation and invasion and improve the adhesion ability of endometrial cancer cells (He et al., 2021). Wen et al. (Wen et al., 2014) have also reported that JAK1/STAT3 plays a crucial role in ovarian cancer as a pro-oncogenic signaling pathway. The targeted inhibition of the JAK1/STAT3 pathway can effectively prevent the progression and metastasis of ovarian cancer. *APC* is a tumor suppressor gene. The frequency of APC germline mutations in patients with familial adenomatous polyposis (FAP)-related diseases, such as gastric fundus adenomatous polyposis, duodenal adenoma, desmoid tumors, and thyroid cancer is greater than 60% (Takao et al., 2021). Enhancer of rudimentary homolog (ERH) is a small, highly conserved protein. It binds to various factors involved in many cellular processes, such as pyrimidine metabolism, mitosis, and transcriptional regulation (Fujimura et al., 2012). The overexpression of ERH weakens the invasion and migration ability of gastric cancer cells, suggesting that it is a prognostic marker (Park et al., 2020). However, a study of ovarian cancer suggested that ERH may be a associated with a poor prognosis, and inhibiting ERH expression can promote cancer cell apoptosis and inhibit the metastasis and invasion of ovarian cancer cells by regulating the epithelial-mesenchymal transition (EMT) (Zhang et al., 2020). Cellular nucleic acid‐binding protein (CNBP) is associated with cell proliferation and is highly expressed in various human tumors. The lncRNA SUMO1P3 enhances proliferation, invasiveness, and drug resistance in gastric cancer cell lines by directly binding to CNBP, resulting in high levels of c-myc and cyclinD1 (CCND1) (Guo et al., 2020). The Bcl-2 family is an important family of apoptosis regulatory proteins, with key roles in the apoptosis signal transduction pathway. Bax is a pro-apoptotic factor in the Bcl-2 family of proteins (Meng et al., 2019). In human retinoblastoma, the expression of anti-apoptotic Bcl-2 is significantly related to poor differentiation and strong invasiveness, and the lack of Bax expression is related to choroidal infiltration and lymph node metastasis (Singh et al., 2015). These previous results indicate that the necroptosis-related genes identified in this study play important roles in various human tumors, further supporting their potential roles in the occurrence and development of NBL. However, further research is needed to explore the molecular mechanisms of action of these genes.

We analyzed the predictive value of the six gene-based model for OS. A Kaplan-Meier analysis showed that OS was significantly lower in the middle-and high-risk groups than in the low-risk group. An ROC curve analysis showed that the prognostic model has good stability and can effectively screen for patients with NBL with poor prognosis. The risk score was identified as an independent prognostic factor for NBL by univariate and multivariate analyses after stratification according to clinical parameters.

Clinical data have shown that immunotherapy with the disialoganglioside GD2 combined with granulocyte-macrophage colony-stimulating factor (GM-CSF) or interleukin-2 significantly improves prognosis in high-risk patients with NBL (Yu et al., 2010). Subsequently, the molecular events associated with NBL-related immune cell infiltration and immune responses in NBL have been a focus of research. In a study of the relationship between immune cell infiltration and prognosis in NBL, Schaafsma et al. (2021) discovered that a high abundance of naïve B cells, memory B cells, CD8^+^ T cells, and NK cells was significantly associated with a longer OS; conversely, high levels of CD4^+^ T cell infiltration were negatively associated with OS. Our results revealed that B cells play an important role in the NBL tumor microenvironment, suggesting that B cells can be used as an independent variable to predict recurrence-free and overall survival. Batchu (Sai, 2020) have shown that low levels of CD4^+^ naïve T cells and monocytes are associated with a reduced event-free survival. Tumor-associated macrophages (TAMs) closely resemble M2-polarized macrophages and are critical modulators of the tumor microenvironment. TAM aggregation in a variety of human tumors is associated with poor clinical outcomes, and TAMs can provide a favorable microenvironment for tumor progression (Liu and Joshi, 2020). TAMs can upregulate the expression of MYC *via* the signal transducer and activator of transcription 3 (STAT3) pathway, and this may explain the association between TAMs and a poor prognosis in patients with non-MYCN-amplified NBL (Liu and Joshi, 2020). In our prognostic model, the relative abundance of plasma cells in the low-risk group was significantly lower than that in the high-risk group, and the risk score was significantly correlated with plasma cells and CD4 memory resting T cells. The six genes in the prognostic model were closely associated with levels of immune cell infiltration and played an important role in the immune microenvironment. These results suggest that necroptosis influences tumorigenesis and tumor development by regulating immune cell infiltration.

We analyzed the specific signaling pathways differentiating the high- and low-risk groups based on the prognostic model. GSVA results showed that the differential pathways between the two groups were mainly signaling pathways such as TGF-beta signaling, mTORC1 signaling, and oxidative phosphorylation. By a GSEA, enriched GO terms were axon extension and dendrite morphogenesis; enriched KEGG pathways were base excision repair, long-term Potentiation, and axon guidance. Transforming growth factor-β (TGF-β) pathway plays an important role in cellular homeostasis by regulating cell growth inhibition, cellular senescence, differentiation, and apoptosis (Lin et al., 2005). The EMT is a transdifferentiation process in which epithelial cells lose polarity and contact inhibition and obtain mesenchymal characteristics, such as the fibroblast migration phenotype. The EMT plays an important role in human embryonic development and is also considered a pathological mechanism. Cancer cells can acquire migration and invasion abilities through the EMT, leading to tumor metastasis. In human NB cell lines, EMT is significantly up-regulated via the TGF-β pathway, resulting in a more aggressive phenotype (Naiditch et al., 2015). MYCN plays an important role in NB. The MYC genes (*MYCN, c-myc,* and *L-myc*) drive tumorigenesis in part by the activation of the mammalian target of rapamycin (mTOR) pathway, a master regulator of translation and protein synthesis. Therefore, the effective inhibition of mTOR function represents a potential therapeutic strategy targeting MYCN in NB (Moreno-Smith et al., 2017). Oxidative phosphorylation (OXPHOS) is an important pathway for the survival and proliferation of tumor cells. Some compounds inhibit the growth of NB *in vivo* by inhibiting the activities of OXPHOS and mitochondrial respiratory complex I (Nagasaki-Maeoka et al., 2020). Base excision repair (BER) fixes the majority of endogenous DNA damage, including deamination, depurination, alkylation, and oxidative damage. Abnormalities in this pathway are strongly associated with tumorigenesis (Wallace et al., 2012). These findings show that the enriched pathways associated with the genes in the prognostic model are closely related to the development of NB.

We also studied the relationship between the risk score obtained by the newly developed model and clinical drug sensitivity. The risk score was significantly correlated with the sensitivity to AS601245, AZD0530, AZD6244, AZD6482, CHIR99021, CCT007093, and other drugs. These chemotherapeutic drugs have been studied extensively in human glioblastoma (GBM). AZD0530, a potent small-molecule inhibitor of Src family kinases, can enhance the radiosensitivity of GBM tumor cells (Yun et al., 2021). AZD6244, an inhibitor of MEK in the RAF/MEK/ERK pathway, inhibits proliferation in the GBM cell line (See et al., 2012). As an inhibitor of PI3Kβ in the PI3K/Akt pathway, AZD6482 can exert an anti-tumor effect by inhibiting proliferation and inducing apoptosis in human GBN tumor cells (Xu et al., 2019). CCT007093 can attenuate cell proliferation, migration, and invasion induced by UVC radiation in human GBM (Yang et al., 2014). The role of these drugs in children with NBL needs to be confirmed by further studies.

The results of this study provide new insights into the occurrence and development of NBL from the perspective of necroptosis. The prognostic model based on six necroptosis-related genes can effectively predict the prognosis of patients with NBL. In addition, the risk score obtained from the necroptosis model is associated with essential biological functions and has clinical value.

## Conclusion

By a variety of bioinformatics analyses of high-throughput sequencing datasets, we systematically evaluated the molecular characteristics and prognostic value of necroptosis in NBL. Our results provide preliminary evidence for the complex biological functions and immunoregulatory effects of necroptosis-related genes. These necroptosis-related genes may be involved in the occurrence, development, invasion, and metastasis of NBL. We constructed a risk score model that can independently predict prognosis in NBL. Our results will aid in revealing the pathogenesis of NBL and in the identification of new biomarkers and provide a basis for the development of therapeutic strategies targeting necroptosis.

## Data Availability

The datasets presented in this study can be found in online repositories. The names of the repository/repositories and accession number(s) can be found in the article/Supplementary Material.

## Abbreviations

GN, Ganglioneuroma; GO, Gene ontology; GSEA, Gene set enrichment analysis; GSVA, Gene set variation analysis; IHC, Immunohistochemistry; KEGG, Kyoto Encyclopedia of Genes and Genomes; LASSO, Least absolute shrinkage and selection operator; NBL, Neuroblastoma; ROC, Receiver operating characteristic.

## References

[B1] Dai X. Hakizimana O. Zhang X. Kaushik A. C. Zhang J. (2020). Orchestrated efforts on host network hijacking: Processes governing virus replication. Virulence 11 (1), 183–198. 10.1080/21505594.2020.1726594 32050846 PMC7051146

[B2] Fujimura A. Kishimoto H. Yanagisawa J. Kimura K. (2012). Enhancer of rudimentary homolog (ERH) plays an essential role in the progression of mitosis by promoting mitotic chromosome alignment. Biochem. Biophys. Res. Commun. 423 (3), 588–592. 10.1016/j.bbrc.2012.06.018 22704934

[B3] Fusco P. Esposito M. R. Tonini G. P. (2018). Chromosome instability in neuroblastoma. Oncol. Lett. 16 (6), 6887–6894. 10.3892/ol.2018.9545 30546420 PMC6256707

[B4] Gong Y. Fan Z. Luo G. Yang C. Huang Q. Fan K. (2019). The role of necroptosis in cancer biology and therapy. Mol. Cancer 18 (1), 100. 10.1186/s12943-019-1029-8 31122251 PMC6532150

[B5] Guo Y. Wang Y. Ma Y. Chen G. Yue P. Li Y. (2020). Upregulation of lncRNA SUMO1P3 promotes proliferation, invasion and drug resistance in gastric cancer through interacting with the CNBP protein. RSC Adv. 10 (10), 6006–6016. 10.1039/c9ra09497k 35497433 PMC9049591

[B6] Hanahan D. Weinberg R. A. (2011). Hallmarks of cancer: The next generation. Cell. 144 (5), 646–674. 10.1016/j.cell.2011.02.013 21376230

[B7] Haq S. Sarodaya N. Karapurkar J. K. Suresh B. Jo J. K. Singh V. (2022). CYLD destabilizes NoxO1 protein by promoting ubiquitination and regulates prostate cancer progression. Cancer Lett. 525, 146–157. 10.1016/j.canlet.2021.10.032 34742871

[B8] He Y. Ma H. Wang J. Kang Y. Xue Q. (2021). miR-20a-5p inhibits endometrial cancer progression by targeting janus kinase 1. Oncol. Lett. 21 (5), 427. 10.3892/ol.2021.12688 33850568 PMC8025135

[B9] Johnstone R. W. Ruefli A. A. Lowe S. W. (2002). Apoptosis: A link between cancer genetics and chemotherapy. Cell. 108 (2), 153–164. 10.1016/s0092-8674(02)00625-6 11832206

[B10] Kobayashi T. Masoumi K. C. Massoumi R. (2015). Deubiquitinating activity of CYLD is impaired by SUMOylation in neuroblastoma cells. Oncogene 34 (17), 2251–2260. 10.1038/onc.2014.159 24909169

[B11] Lin H. K. Bergmann S. Pandolfi P. P. (2005). Deregulated TGF-beta signaling in leukemogenesis. Oncogene 24 (37), 5693–5700. 10.1038/sj.onc.1208923 16123802

[B12] Liu K. X. Joshi S. (2020). "Re-educating" tumor associated macrophages as a novel immunotherapy strategy for neuroblastoma. Front. Immunol. 11, 1947. 10.3389/fimmu.2020.01947 32983125 PMC7493646

[B13] Meng K. Yuan G. Bao H. Wang L. Ma R. Yu B. (2019). Interaction of HCCR-1 and Bax in breast cancer. J. BUON 24 (3), 1027–1037. 31424657

[B14] Moreno-Smith M. Lakoma A. Chen Z. Tao L. Scorsone K. A. Schild L. (2017). p53 nongenotoxic activation and mTORC1 inhibition lead to effective combination for neuroblastoma therapy. Clin. Cancer Res. 23 (21), 6629–6639. 10.1158/1078-0432.CCR-17-0668 28821555 PMC5959272

[B15] Nagasaki-Maeoka E. Ikeda K. Takayama K. I. Hirano T. Ishizuka Y. Koshinaga T. (2020). Polyethylene glycol derivative 9bw suppresses growth of neuroblastoma cells by inhibiting oxidative phosphorylation. Cancer Sci. 111 (8), 2943–2953. 10.1111/cas.14512 32495467 PMC7419032

[B16] Naiditch J. A. Jie C. Lautz T. B. Yu S. Clark S. Voronov D. (2015). Mesenchymal change and drug resistance in neuroblastoma. J. Surg. Res. 193 (1), 279–288. 10.1016/j.jss.2014.07.018 25128389

[B17] Najafov A. Chen H. Yuan J. (2017). Necroptosis and cancer. Trends Cancer 3 (4), 294–301. 10.1016/j.trecan.2017.03.002 28451648 PMC5402749

[B18] Negroni A. Colantoni E. Cucchiara S. Stronati L. (2020). Necroptosis in intestinal inflammation and cancer: New concepts and therapeutic perspectives. Biomolecules 10 (10), 1431. 10.3390/biom10101431 PMC7599789 33050394

[B19] Park J. H. Park M. Park S. Y. Lee Y. J. Hong S. C. Jung E. J. (2020). ERH overexpression is associated with decreased cell migration and invasion and a good prognosis in gastric cancer. Transl. Cancer Res. 9 (9), 5281–5291. 10.21037/tcr-20-1498 35117894 PMC8797358

[B20] Park J. R. Eggert A. Caron H. (2010). Neuroblastoma: Biology, prognosis, and treatment. Hematol. Oncol. Clin. North Am. 24 (1), 65–86. 10.1016/j.hoc.2009.11.011 20113896

[B21] Sai B (2020). Immunological landscape of Neuroblastoma and its clinical significance[J]. Cancer Treat. Res. Commun. 26, 100274. 10.1016/j.ctarc.2020.100274 33338852

[B22] Schaafsma E. Jiang C. Cheng C. (2021). B cell infiltration is highly associated with prognosis and an immune-infiltrated tumor microenvironment in neuroblastoma. J. Cancer Metastasis Treat. 7 (34). 10.20517/2394-4722.2021.72 PMC8389852 34458583

[B23] See W. L. Tan I. L. Mukherjee J. Nicolaides T. Pieper R. O. (2012). Sensitivity of glioblastomas to clinically available MEK inhibitors is defined by neurofibromin 1 deficiency. Cancer Res. 72 (13), 3350–3359. 10.1158/0008-5472.CAN-12-0334 22573716 PMC4128256

[B24] Singh L. Pushker N. Saini N. Sen S. Sharma A. Bakhshi S. (2015). Expression of pro-apoptotic Bax and anti-apoptotic Bcl-2 proteins in human retinoblastoma. Clin. Exp. Ophthalmol. 43 (3), 259–267. 10.1111/ceo.12397 25132102

[B25] Takao M. Yamaguchi T. Eguchi H. Yamada T. Okazaki Y. Tomita N. (2021). APC germline variant analysis in the adenomatous polyposis phenotype in Japanese patients. Int. J. Clin. Oncol. 26 (9), 1661–1670. 10.1007/s10147-021-01946-4 34106356

[B26] Wallace S. S. Murphy D. L. Sweasy J. B. (2012). Base excision repair and cancer. Cancer Lett. 327 (1-2), 73–89. 10.1016/j.canlet.2011.12.038 22252118 PMC3361536

[B27] Wen W. Liang W. Wu J. Kowolik C. M. Buettner R. Scuto A. (2014). Targeting JAK1/STAT3 signaling suppresses tumor progression and metastasis in a peritoneal model of human ovarian cancer. Mol. Cancer Ther. 13 (12), 3037–3048. 10.1158/1535-7163.MCT-14-0077 25319391 PMC4321961

[B28] Xu P. F. Yang J. A. Liu J. H. Yang X. Liao J. M. Yuan F. E. (2019). PI3Kβ inhibitor AZD6482 exerts antiproliferative activity and induces apoptosis in human glioblastoma cells. Oncol. Rep. 41 (1), 125–132. 10.3892/or.2018.6845 30542720 PMC6278584

[B29] Yang L. Zhou Z. Yao D. Xu W. Zhao H. (2014). ET-69 * specific wip1 inhibitor, cct007093 abrogate cell proliferation, migration and invasion induced by the uvc radiation in human glioblastoma cells. Neuro. Oncol. 16 (5), v94. 10.1093/neuonc/nou255.66

[B30] Yu A. L. Gilman A. L. Ozkaynak M. F. London W. B. Kreissman S. G. Chen H. X. (2010). Anti-GD2 antibody with GM-CSF, interleukin-2, and isotretinoin for neuroblastoma. N. Engl. J. Med. 363 (14), 1324–1334. 10.1056/NEJMoa0911123 20879881 PMC3086629

[B31] Yun H. S. Lee J. Kil W. J. Kramp T. R. Tofilon P. J. Camphausen K. (2021). The radiosensitizing effect of AZD0530 in glioblastoma and glioblastoma stem-like cells. Mol. Cancer Ther. 20 (9), 1672–1679. 10.1158/1535-7163.MCT-20-0883 34158343 PMC8419151

[B32] Zhang D. Chu Y. J. Song K. J. Chen Y. L. Liu W. Lv T. (2020). Knockdown of enhancer of rudimentary homolog inhibits proliferation and metastasis in ovarian cancer by regulating epithelial-mesenchymal transition. Biomed. Pharmacother. 125, 109974. 10.1016/j.biopha.2020.109974 32036222

